# Selection and Validation of Reference Genes for RT-qPCR Analysis in *Spinacia oleracea* under Abiotic Stress

**DOI:** 10.1155/2021/4853632

**Published:** 2021-02-03

**Authors:** Hao Xie, Bo Li, Yu Chang, Xiaoyan Hou, Yue Zhang, Siyi Guo, Yuchen Miao, Quanhua Wang, Sixue Chen, Yinghua Su, Ying Li, Shaojun Dai

**Affiliations:** ^1^Key Laboratory of Saline-Alkali Vegetation Ecology Restoration (Northeast Forestry University), Ministry of Education, College of Life Sciences, Northeast Forestry University, Harbin 150040, China; ^2^Development Center of Plant Germplasm Resources, College of Life Sciences, Shanghai Normal University, Shanghai 200234, China; ^3^Institute of Plant Stress Biology, State Key Laboratory of Cotton Biology, Department of Biology, Henan University, Kaifeng 455000, China; ^4^Department of Biology, Genetics Institute, Plant Molecular and Cellular Biology Program, Interdisciplinary Center for Biotechnology Research, University of Florida, Gainesville, FL 32610, USA; ^5^State Key Laboratory of Crop Biology, College of Life Sciences, Shandong Agricultural University, Tai'an, 271018 Shandong, China

## Abstract

Reverse transcription quantitative real-time polymerase chain reaction (RT-qPCR) is an accurate and convenient method for mRNA quantification. Selection of optimal reference gene(s) is an important step in RT-qPCR experiments. However, the stability of housekeeping genes in spinach (*Spinacia oleracea*) under various abiotic stresses is unclear. Evaluating the stability of candidate genes and determining the optimal gene(s) for normalization of gene expression in spinach are necessary to investigate the gene expression patterns during development and stress response. In this study, ten housekeeping genes, *18S ribosomal RNA* (*18S rRNA*), *actin*, *ADP ribosylation factor* (*ARF*), *cytochrome c oxidase* subunit 5C (*COX*), *cyclophilin* (*CYP*), *elongation factor 1-alpha* (*EF1α*), *glyceraldehyde-3-phosphate dehydrogenase* (*GAPDH*), *histone H3* (*H3*), *50S ribosomal protein L2* (*RPL2*), and *tubulin alpha chain* (*TUBα*) from spinach, were selected as candidates in roots, stems, leaves, flowers, and seedlings in response to high temperature, CdCl_2_, NaCl, NaHCO_3_, and Na_2_CO_3_ stresses. The expression of these genes was quantified by RT-qPCR and evaluated by NormFinder, BestKeeper, and geNorm. *18S rRNA*, *actin*, *ARF*, *COX*, *CYP*, *EF1α*, *GAPDH*, *H3*, and *RPL2* were detected as optimal reference genes for gene expression analysis of different organs and stress responses. The results were further confirmed by the expression pattern normalized with different reference genes of two heat-responsive genes. Here, we optimized the detection method of the gene expression pattern in spinach. Our results provide the optimal candidate reference genes which were crucial for RT-qPCR analysis.

## 1. Introduction

Reverse transcription quantitative real-time polymerase chain reaction (RT-qPCR) is an accurate and convenient method of quantifying mRNA levels of gene expression [1]. Selection of appropriate reference genes is crucial for validating accurate gene expression [2, 3]. Improper reference genes used in data processing may lead to inaccurate and even wrong results [4]. The commonly used reference genes in RT-qPCR analysis are the housekeeping genes because they are usually expressed steadily at mRNA levels in any organs under various conditions [5, 6]. Although *18S ribosomal RNA* (*18S rRNA*), *actin*, and *glyceraldehyde-3-phosphate dehydrogenase* (*GAPDH*) are usually taken as reference genes [5], their mRNAs are not always stable in any cases [7–10]. Therefore, selection and optimization of reference genes are important steps in RT-qPCR experiments.

Some statistical algorithms, such as BestKeeper, NormFinder, and geNorm, are widely used for analyses of reference genes for RT-qPCR. BestKeeper analyzes the stability by calculating the standard deviation (SD) of the quantification cycle (Cq) values of candidate reference genes [11]. NormFinder compares the variation within and between sample groups of candidate genes and calculates the stability value of each gene based on the 2^−ΔCq^ of genes [12]. geNorm evaluates the stability of candidate genes through calculating the stability value based on the geometric mean of 2^−ΔCq^ of genes and mean pairwise variation in sample groups, as well as providing the optimal numbers of reference genes under each condition [13].

Spinach (*Spinacia oleracea*) belongs to the Amaranth family and is rich in carotenoid, vitamins, and minerals. It is a favorite vegetable all over the world. In our previous study, the draft of the spinach genome has been sequenced, with 25,495 encoding genes predicted [14]. Moreover, the patterns of gene expression and protein abundance have been reported in spinach in response to diverse stresses (e.g., heat, salinity, heavy metal, and virus) using molecular genetic, transcriptomic, and proteomic approaches [15–23].

To date, there are only a few reports on gene characterization and function analyses in spinach in response to stresses. A recent study reported that the *S. oleracea heat shock 70* (*SoHSC70*) was induced by heat stress, and overexpression of *SoHSC70* enhanced the heat tolerance in spinach calli [24]. Besides, spinach *cytochrome P450 85A1* (*SoCYP85A1*) was upregulated in response to *Phytophthora nicotianae* infection, and 35S-promoted *SoCYP85A1* overexpression conferred resistance to *P. nicotianae* pathogen inoculation in tobacco [25]. In addition, the spinach nonsymbiotic hemoglobin family gene (*SoHb*) was induced by several stress treatments (i.e., polyethylene glycol, NaCl, H_2_O_2_, salicylic acid, and nitric oxide) but suppressed by a nitric oxide scavenger, nitrate reductase inhibitor, and nitric oxide synthase inhibitor. Overexpression of *SoHb* in *Arabidopsis* resulted in the decreases in nitric oxide level and sensitivity to nitrate stress [26]. In these studies, some housekeeping genes in spinach were used as reference genes in RT-qPCR experiments for normalization analysis. *18S rRNA* was used as a reference gene to detect the expression patterns of chilling-/drought-responsive *SoCAP85* (85 kD *cold acclimation protein*) [27], drought-/salt-/oxidative stress-responsive *SoHb* [26], 13 heat-responsive genes (including *SoHSFB2b* and *SobZIP9*) [22], 15 nitrate transport and assimilation-related genes [28], and anthocyanin biosynthesis-related genes in various spinach germplasms [29]. In addition, *actin*, *GAPDH*, and *ubiquitin 5* (*UBQ5*) were used to normalize the expression levels of several genes in response to various stresses, such as drought [30], biotic stress [25, 31], gibberellic acid (GA) treatment, and gender-specific condition [32]. However, only one study evaluated the stability of these reference genes [27]. Five commonly used housekeeping genes (i.e., *GAPDH*, *actin*, *16S rRNA*, *tubulin alpha chain* (*TUBα*), and *18S rRNA*) were evaluated between partially/fully hydrated versus dry seeds of spinach under chilling, desiccation, and optimum conditions. Among them, *18S rRNA* appeared to be most stable, but still fluctuated under several treatments [27].

Some other housekeeping genes, such as *ADP ribosylation factor* (*ARF*), *cytochrome c oxidase* (*COX*), *cyclophilin* (*CYP*), *elongation factor 1-alpha* (*EF1α*), *histone H3* (*H3*), and *50S ribosomal protein L2* (*RPL2*), were widely used as reference genes for RT-qPCR analysis in many plants [33–40]. *ARF*s encode small GTP-binding proteins (GTPases) for regulating various biological processes (e.g., cell division, cell expansion, and cellulose biosynthesis) [41–43], and COX is the terminal oxidase for mitochondrial oxidative metabolism and ATP synthesis [44]. CYP is critical in facilitating protein folding, which is involved in diverse cellular processes, such as apoptosis [45], immune response [46], and spliceosome assembly [47]. In addition, the histones and RPL are the components of the chromosome and ribosome, respectively [48, 49], and EF1*α* is involved in the protein synthesis [50]. However, there is still little evaluation on these reference genes in spinach.

In this study, ten candidate genes (i.e., *18S rRNA*, *actin*, *ARF*, *COX*, *CYP*, *EF1α*, *GAPDH*, *H3*, *RPL2*, and *TUBα*) were selected for reference genes in spinach. Three Excel programs (i.e., BestKeeper, geNorm, and NormFinder) were used to evaluate the stability of these candidate genes in different organs, as well as stresses of heat, heavy metal, NaCl, Na_2_CO_3_, and NaHCO_3_. Optimal reference genes for each condition were verified. In addition, the two stable reference genes, *ARF* and *actin*, and the commonly used reference gene *TUBα* were selected to normalize the mRNA levels of two representative heat-responsive genes (*SobZIP9* and *SoHSFB2b*) on references.

## 2. Materials and Methods

### 2.1. Plant Materials and Growth Condition

A heat-resistant sibling inbred line of spinach, Sp75, was used in this study. Spinach plants were placed in a growth chamber with a temperature regime of 22/18°C, 10/14 h day/night cycle, and a relative humidity of 60%. The top third and fourth leaves, stems, roots, male flowers, and female flowers of plants with uniform growth were sampled at 50 days after planting. Seedlings were sampled at 10 days after planting. These samples were flash-frozen in liquid nitrogen and stored at -80°C for further experiment. Three biological replicates were taken for each organ, and at least three plants were used for each replicate.

### 2.2. Stress Treatment

Fifty-day-old spinach seedlings with uniform growth were used for stress treatment. For heat treatments, the plants were moved into a chamber (37°C) at 0, 1, 2, 4, 6, 8, 12, 24, and 48 h before they were sampled [15]. For the treatments with heavy metal, salt, and alkali, the seedlings were watered with 200 *μ*mol/L CdCl_2_ [51], 200 mmol/L NaCl [52], 200 mmol/L NaHCO_3_ [53], and 100 mmol/L Na_2_CO_3_ [54, 55], and the treatment times are 0, 1, 3, 6, 12, 24, and 48 h [56]. The top third and fourth leaves as well as roots were sampled for RNA extraction. Three biological replicates were taken for each time point of all stress treatments, and at least three plants were used for each replicate.

### 2.3. RNA Isolation and First-Strand cDNA Synthesis

Plant samples were ground in liquid nitrogen with a mortar and a pestle. Total RNA was isolated from 100 mg sample powder with TRIzol™ LS Reagent (Invitrogen, USA). The RNA samples with 260/280 ratios ranging from 1.8 to 2.1 were used for the following experiment. Total RNA was also examined by electrophoresis with 1% agarose gel to ensure the integrity. One microgram of total RNA was used for first-strand cDNA synthesis in a 20 *μ*L total volume with a mixture of oligo dT primer and Random 6-mer in PrimeScript™ RT reagent (Takara, Japan) according to the manufacturer's instructions.

### 2.4. Candidate Reference Gene Selection

Ten candidate genes (*18S rRNA*, *actin*, *ARF*, *COX*, *CYP*, *EF1α*, *GAPDH*, *H3*, *RPL2*, and *TUBα*) were selected for this study according to their homologous gene stability in other plant species, such as sweet potato (*Ipomoea batatas*) [38], radish (*Raphanus sativus*) [57], potato (*Solanum tuberosum*) [39], soybean (*Glycine max*) [40], kenaf (*Hibiscus cannabinus*) [34], corn poppy (*Papaver rhoeas*) [37], bladder campion (*Silene vulgaris*) [36], *Achyranthes bidentata* [35], and *Baphicacanthus cusia* [33]. The sequences of these candidate homologous genes in spinach were obtained from SpinachBase (http://www.spinachbase.org) [14, 58, 59].

### 2.5. Primer Design and Evaluation

The primer pairs of each reference gene were designed according to their sequences by using the online program Primer3Plus (Table 1) (http://www.primer3plus.com/cgi-bin/dev/primer3plus.cgi) [60]. The mixed cDNA from all samples was used as a template in primer evaluation. The PCR amplification products of each primer pair were checked by 2% agarose gel electrophoresis and sequencing, and then, the specificity of each pair of primers was evaluated by melting curve analysis followed by the amplification in RT-qPCR. Standard curves of each primer pair were established using a 5-fold dilution series ([1/1], [1/5], [1/25], [1/125], [1/625], and [1/3125]) of template cDNA [61]. The amplification efficiencies (*E*) of these primer pairs were calculated by the slope of standard curves (*E* = 10 − 1/slope), and the correlation coefficients (*R*^2^) were acquired from the standard curves as well [62].

### 2.6. Real-Time Quantitative PCR

RT-qPCR analysis was performed in 0.2 mL tubes with the Applied Biosystems 7500 Real-Time PCR System (ABI, USA). Each reaction contained 1 *μ*L cDNA (5-time diluted), 10 *μ*L AceQ Universal SYBR qPCR Master Mix (Vazyme, China), 0.5 *μ*L of primer (500 *μ*mol/L), and 8 *μ*L deionized water. The PCR was carried out as the following program: predegeneration at 95°C for 3 min; 40 cycles of degeneration at 95°C for 15 s, annealing at 55°C for 15 s, and extension at 72°C for 30 s; and melting curve analysis at 65°C-95°C. RT-qPCR of each cDNA sample was carried out three times as technical replicates.

### 2.7. Stability Evaluation of Candidate Reference Genes

The Cq values of 10 candidate genes were put in an Excel sheet, and a boxplot of these Cq values was generated. The stability of these 10 genes was evaluated by three statistical Excel macro programs, including BestKeeper [11], NormFinder [12], and geNorm [13]. The Cq values of each gene in roots, stems, leaves, flowers, and seedlings as well as leaves and roots in response to various stresses (i.e., high temperature, NaCl, Na_2_CO_3_, NaHCO_3_, and CdCl_2_) were used in this evaluation. In BestKeeper, the standard deviation (SD) of the Cq values of each candidate gene was calculated, and the gene with the lowest SD was taken as the most stable gene [11, 63]. In NormFinder, the expression stability (M1) was calculated by Cq values obtained by RT-qPCR of candidate genes and ranked in each sample set. The lowest M1 indicates that the gene is most stable [12]. In geNorm, the stability of candidate genes was evaluated by relative expression levels (*Q*) transformed from the Cq values for each sample according to the formula of *Q* = 2^−ΔCq^(ΔCq = Cq value of each sample − the minimum Cq value in each set). This formula works under the precondition that the efficiency of primers should range from 90% to 105% [64, 65]. An average expression stability value (M2) of each candidate was calculated to demonstrate their stability. The gene with the lowest M2 value was regarded as the most stable expression. Besides, the geNorm software also determines the optimal number of reference genes required for RT-qPCR data normalization under each condition by pairwise variation (*V*_*n*_/*V*_*n*+1_) between the normalization factors NF_*n*_ and NF_*n*+1_. If *V*_*n*_/*V*_*n*+1_ < 0.15, the first *n* is the optimal number of genes required for this condition [13].

### 2.8. Normalization of SobZIP9 and SoHSFB2b

The expression pattern of two heat-responsive genes *SobZIP9* and *SoHSFB2b* [22] in spinach was detected in response to heat stress (37°C). The mRNA levels of *SobZIP9* and *SoHSFB2b* were then normalized by the most stable genes suggested by NormFinder and BestKeeper, as well as *TUBα*, a commonly used reference gene. Primer pairs for *SobZIP9* (qSoHSFB2b-F: TCTTTCCACACTCGCTCTGT, qSoHSFB2b-R: CGGATTACAAGAAGGCAGGC) and *SoHSFB2b* (qSobZIP9-F: TGCTGGAAACCCTAGGACTG, qSobZIP9-R: CTTCTGGTGCTTCTAGGCCT) [22] were used in this experiment. A linear ANOVA was used for evaluation of the variation of each gene in response to heat stress.

## 3. Results

### 3.1. Analysis of Primer Specificity and PCR Amplification

**Figure 1:**
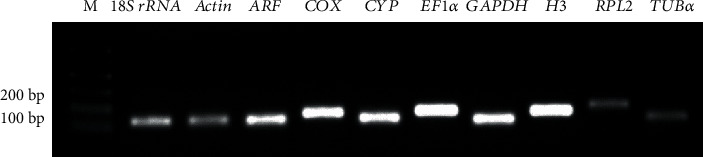
Specificity of primers for ten candidate reference genes in PCR and their amplicon sizes. The names of ten candidate reference genes including *18S ribosomal RNA* (*18S rRNA*), *actin*, *ADP ribosylation factor* (*ARF*), *cytochrome c oxidase* (*COX*), *cyclophilin* (*CYP*), *elongation factor 1-alpha* (*EF1α*), *glyceraldehyde-3-phosphate dehydrogenase* (*GAPDH*), *histone H3* (*H3*), *50S ribosomal protein L2* (*RPL2*), and *tubulin alpha chain* (*TUBα*) are noted on each lane. M represents 100 DNA ladder.

Ten candidates were chosen as reference genes for RT-qPCR analysis. They are *18S rRNA*, *actin*, *ARF*, *COX*, *CYP*, *EF1α*, *GAPDH*, *H3*, *RPL2*, and *TUBα*. To evaluate the specificity of designed primers for the ten genes, the analyses of PCR, gel electrophoresis, and melting curves were performed. The gel electrophoresis showed a single band with expected size of each pair of primers (Figure 1), and the melting curves of each primer pair exhibited a single peak (Supplementary Figure S1), indicating the specificity of these primer pairs of candidate genes. The target amplicons were sequenced, and the results were consistent with their gene sequences in SpinachBase [14, 30, 58]. The standard curves indicated that the RT-qPCR amplification efficiency of candidate genes ranged from 92.53% (*ARF*) to 102.80% (*EF1α*), and the correlation coefficients varied from 0.991 (*COX*) to 0.999 (*actin*, *CYP*, and *GAPDH*) (Table 1 and Supplementary Figure S2). Thus, these primers are specific for their respective genes and can be used in RT-qPCR analysis.

### 3.2. Expression Profiles of Ten Candidate Genes in Spinach

**Figure 2:**
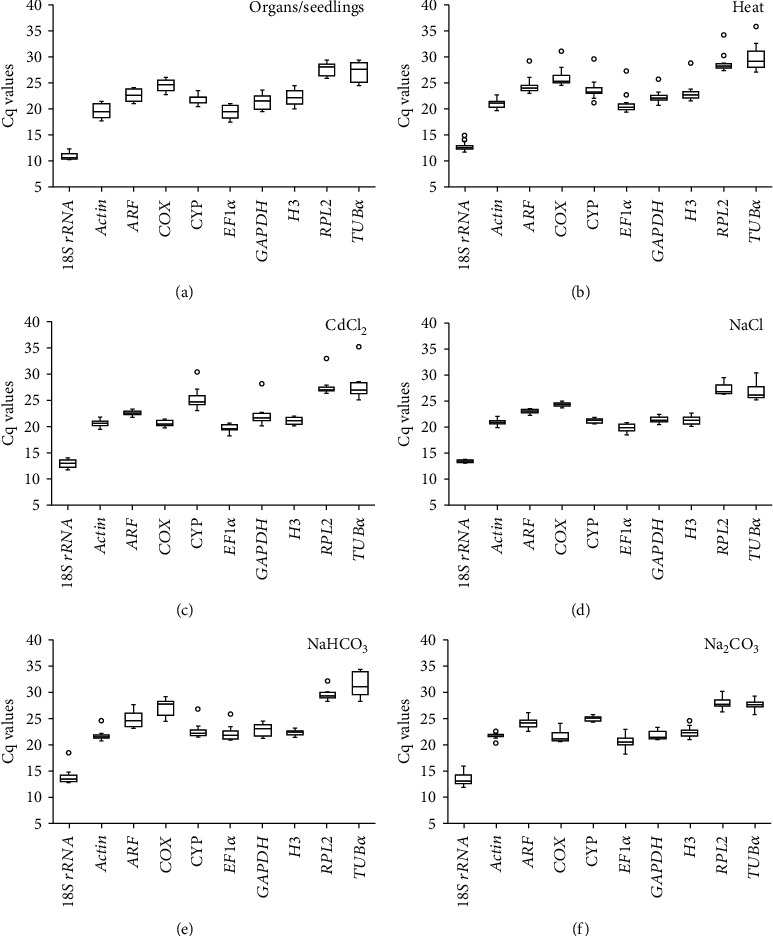
Cq values of ten candidate reference genes in spinach detected by RT-qPCR. The medians of Cq values for these genes are represented by the line in the boxes, and the upper and lower quartiles of Cq values are represented by the upper and lower boundaries of the boxes. The whiskers represent the ranges for the bottom 25% and the top 25% of these Cq values. Small circles represent outliers of these Cq values: (a) Cq values in organs including leaves, stems, roots, flowers, and seedlings, (b) Cq values in the leaves and roots under heat stress, (c) Cq values in the leaves and roots under heavy metal stress, (d) Cq values in the leaves and roots under NaCl, (e) Cq values in the leaves and roots under NaHCO_3_, and (f) Cq values in the leaves and roots under Na_2_CO_3_. Ten candidate reference genes refer to Figure 1. Three biological replicates were taken for different organs/seedlings, as well as leaves and roots at each time point of all stress treatments.

Analysis of the expression levels of ten candidates was performed in young seedlings, roots, stems, leaves, male flowers, and female flowers, as well as leaves and roots in response to stresses of heat, heavy metal, NaCl, Na_2_CO_3_, and NaHCO_3_. Cq values of ten candidates in various samples obtained by RT-qPCR were shown in a boxplot (Figure 2). The Cq values varied from 10.19 (*18S rRNA* in organs/seedlings) to 35.79 (*TUBα* in heat treatment) indicating that these candidate genes present different expression levels. The Cq value range reveals variability among the candidate genes. *18S rRNA* shows the minimal range of Cq values in organs/seedlings (2.08, Figure 1(a)) and under NaCl treatment (0.86, Figure 1(d)), while *actin* (3.07, Figure 1(c)), *ARF* (1.64, Figure 1(c)), *GAPDH* (3.09, Figure 1(e)), and *CYP* (1.38, Figure 1(f)) show the minimal range of Cq values under heat, heavy metal (CdCl_2_), NaHCO_3_, and Na_2_CO_3_ treatments, respectively. These minimal ranges of Cq values indicated that these genes are more stable than others in each condition. However, further analyses are needed, because the comparison of the range of Cq values along is deficient to reveal the stability of the candidate genes. Thus, statistical macro programs were then used in this study.

### 3.3. Expression Stability of Ten Candidate Genes

**Figure 3:**
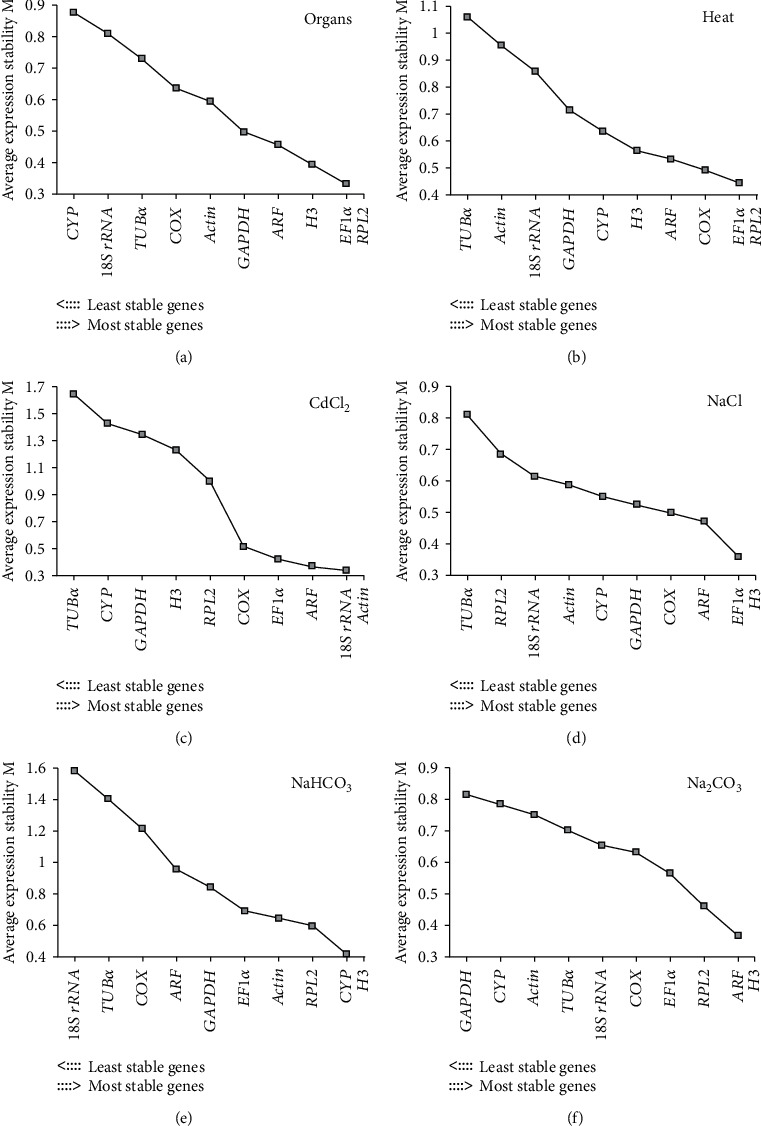
Average expression stability values (M2) in different data sets obtained from the software geNorm. The stability values (M2) and stability rank were obtained from geNorm. A lower M2 value suggests higher stability: (a) stability rank obtained in organs including leaves, stems, roots, flowers, and seedlings, (b) stability rank obtained in the leaves and roots under heat stress, (c) stability rank obtained in the leaves and roots under heavy metal stress, (d) stability rank obtained in the leaves and roots under NaCl, (e) stability rank obtained in the leaves and roots under NaHCO_3_, and (f) stability rank obtained in the leaves and roots under Na_2_CO_3_. Ten candidate reference genes refer to Figure 1. Three biological replicates were taken for different organs/seedlings, as well as leaves and roots at each time point of all stress treatments.

**Figure 4:**
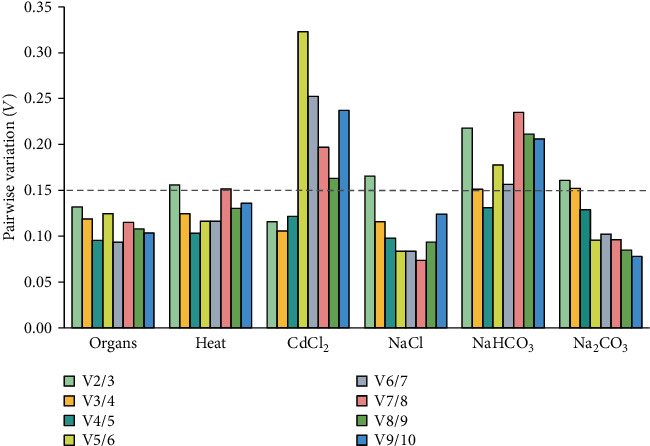
Pairwise variation (*V*) analysis of the candidate reference genes. The geNorm software was used to analyze the pairwise variation (*V*_*n*_/*V*_*n*+1_) between the normalization factors (NF) NF_*n*_ and NF_*n*+1_ in order to determine the optimal number of candidate reference genes required for RT-qPCR data normalization. If *V*_*n*_/*V*_*n*+1_ < 0.15 (gray dotted line), the minimum value of *n* is the optimal number of genes required.

Three statistical Excel macro programs (i.e., BestKeeper, NormFinder, and geNorm) were used to evaluate the stability of ten candidate genes, in order to find optimal reference genes in spinach for RT-qPCR normalization (Table 2, Figures 3 and 4). The stability of candidate genes was evaluated in 6 data sets, organs/seedlings, leaves, and roots under heat, heavy metal, NaCl, NaHCO_3_, and Na_2_CO_3_, respectively.

The stability of candidate genes was analyzed with BestKeeper (Table 2), which can calculate the standard deviation (SD) on the basis of the Cq values of all candidate reference genes [11]. The reference genes exhibiting the lowest standard deviation (SD) were taken as the most stable genes [63]. In the samples of different organs, *18S rRNA* (SD = 0.58) and *CYP* (SD = 0.72) were regarded as the optimal reference genes. Under the heat (37°C) treatment, the expression levels of *actin* (SD = 0.57) and*18S rRNA* (SD = 0.60) were more stable than those of other genes. Under heavy metal (200 *μ*M CdCl_2_) treatment, *ARF* (SD = 0.37) and *COX* (SD = 0.49) displayed stable expression. Under NaCl (200 mM) treatment, *18S rRNA* (SD = 0.24) and *ARF* (SD = 0.32) showed shared and stable expression. *Actin* (SD = 0.64) and *RPL2* (SD = 0.74), as well as *CYP* (SD = 0.24) and *actin* (SD = 0.32), were the stable genes in 200 mM NaHCO_3_ and 100 mM NaCO_3_ treatments, respectively, while *18S rRNA* (SD = 1.03) was the least stable gene under 100 mM NaCO_3_ treatment (Table 2). Importantly, the *TUBα* was identified as the least stable gene in organs (SD = 1.58), as well as under treatments of heat (SD = 1.67), NaCl (SD = 1.24), and NaHCO_3_ (SD = 2.13).

The expression stability of ten genes was also analyzed using the NormFinder software (Table 2), which can provide a stability value (M1) for each gene by comparing the variation within and between sample groups. For the organs/seedlings, there were six sample groups, including young seedlings, roots, stems, leaves, male flowers, and female flowers. For the heat-treated samples of leaves and roots, there were nine sample groups, which were samples under heat (37°C) stresses for 0, 1, 2, 4, 6, 8, 12, 24, and 48 h, respectively. In addition, for the samples under heavy metal, NaCl, Na_2_CO_3_, and NaHCO_3_ treatment, there were six sample groups for each treatment (i.e., 0, 1, 3, 6, 12, 24, and 48 h) and totally 24 sample groups. The variation of each candidate gene in each case was compared, respectively. The gene stability was evaluated by M1 values.

The gene with lower M1 indicated that it was more stable [12]. For the samples from different organs, *ARF* (M1 = 0.131) and *EF1α* (M1 = 0.228) were regarded as the optimal reference genes, while *CYP* (M1 = 0.703) was the least stable gene. Under the heat (37°C) treatment, the expression of *ARF* (M1 = 0.212) and *RPL2* (M1 = 0.228) was more stable than that of others, while *TUBα* (M1 = 0.928) was the least stable gene. Under heavy metal (200 *μ*M CdCl_2_) treatment, *EF1α* (M1 = 0.625) and *RPL2* (M1 = 0.650) were suggested to normalize the expression level of other genes, and *TUBα* (M1 = 1.621) seemed to be inappropriate as a reference gene. Under NaCl (200 mM) treatment, *COX* (M1 = 0.201) and *ARF* (M1 = 0.213) showed the common and highest stable expression, and *TUBα* (M1 = 0.852) was considered the least stable gene. In addition, *RPL2* (M1 = 0.253) and *actin* (M1 = 0.454), as well as *ARF* (M1 = 0.284) and *H3* (M1 = 0.297) were stable genes, but *18S rRNA* (M1 = 1.407) and *GAPDH* (M1 = 0.523) were the least stable genes under NaHCO_3_ and Na_2_CO_3_ treatments, respectively (Table 2).

In addition, geNorm was used for gene stability analysis. geNorm evaluates the stability of candidate genes based on the geometric mean of these genes and mean pairwise variation in sample groups [13]. As mentioned above, there were six, nine, and 24 sample groups for organs/seedlings, heat stress, and other stresses, respectively. The stability value (M2) (Figure 3) and pairwise variation (*V*_*n*_/*V*_*n*+1_) (Figure 4) in the results given by geNorm revealed the gene stability and optimal gene numbers in certain case. The genes with the lowest M2 values were regarded as the most stable ones in each case (Figure 3). Besides, when pairwise variation (*V*_*n*_/*V*_*n*+1_) was less than 0.15, the minimum value of *n* was the optimal number of genes required for such condition (Figure 4). And the suitable gene pair included the genes from rank 1 to rank *n* in each condition [13]. According to this validation method, we recommended several reference genes for RT-qPCR analyses in organs and stress response. In different organs, *EF1α*/*RPL2* (*V*_2_/*V*_3_ = 0.132) was a suitable gene pair for mRNA level normalization (Figures 3(a) and 4). *EF1α*, *RPL2*, and *COX* (*V*_3_/*V*_4_ = 0.125) were identified as an appropriate gene set under heat treatment (Figures 3(b) and 4). Besides, the gene pair of *18S rRNA*/*actin* (*V*_2_/*V*_3_ = 0.116) was recommended for heavy metal stress (200 *μ*M CdCl_2_) (Figures 3(c) and 4), while *EF1α*/*H3*/*ARF* (*V*_3_/*V*_4_ = 0.106) were suggested as reference genes under NaCl (200 mM) (Figures 3(d) and 4) treatment. In addition, *CYP*/*H3*/*RPL2*/*actin* (*V*_4_/*V*_5_ = 0.131) and *ARF*/*H3*/*RPL2*/*EF1α* (*V*_4_/*V*_5_ = 0.129) were selected as reference gene sets under 200 mM NaHCO_3_ (Figures 3(e) and 4) and 100 mM Na_2_CO_3_ (Figures 3(f) and 4), respectively.

### 3.4. Validation Test of Candidate Genes

**Figure 5:**
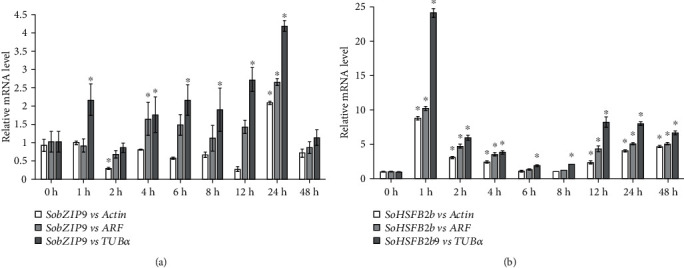
Expression pattern of *SobZIP9* and *S*o*HSFB2b* normalized by different genes in response to heat stress. Expression patterns of two heat-induced genes, *basic region-leucine zipper 9* (*bZIP9*, a) and *heat stress transcription factor 2 b* (*HSFB2b*, b), were normalized with *actin*, *ADP ribosylation factor* (*ARF*), and *tubulin alpha chain* (*TUBα*). Three biological replicates were taken for leaves at each time point of heat treatments. Data represent average ± SD, and ∗ indicates significant difference (*p* < 0.05).

To prove the feasibility of reference genes for RT-qPCR in spinach, mRNA levels of two heat-responsive genes, spinach *basic region-leucine zipper 9* (*SobZIP9*) and *heat stress transcription factor 2b* (*SoHSFB2b*) [22], were detected in spinach under heat treatment and normalized by heat-stable *actin* and *ARF*, as well as commonly used *TUBα*. Under heat stress, the expression level of *SobZIP9* was reduced in spinach after 2-12 h heat treatment when normalized by *actin* and also reduced after 2 h heat treatment when normalized by *ARF*. However, when normalized by *TUBα*, it exhibited an increase under heat treatment (Figure 5(a)). Besides, *SoHSFB2b* was increased about 10-fold after 1 h heat stress normalized by *actin* and *ARF*, but it exhibited 24-time increase when being normalized by *TUBα* (Figure 5(b)). This implies that the *SoHSFB2b* expression level was overestimated when normalized by *TUBα*. In addition, when normalized by *actin* and *ARF*, the levels of heat-reduced *SobZIP9* and heat-induced *SoHSFB2b* were consistent with those in spinach under 35°C for 30 min and 5 h based on results from a previous transcriptomic study [22], which showed that *actin* and *ARF* are the appropriate reference genes in spinach for heat response analysis.

## 4. Discussion

Diverse PCR approaches have been applied in the evaluation of gene expression levels, such as semiquantitative PCR, RT-qPCR, and digital PCR (dPCR). About ten years ago, digital PCR (dPCR) was developed as a novel technology for mRNA quantitation at a single molecular level [66, 67]. The dPCR can detect absolute copy numbers of certain gene expression without reference genes and standard curve [68], and it is more sensitive than qPCR for detecting the smaller copy number variation of genes [69]. However, dPCR is labour-intensive and expensive and has relatively low throughput when compared with qPCR [67], which leads to being not popularly used for the evaluation of gene expression in plant molecular labs. RT-qPCR is a convenient and accurate method to detect the mRNA levels of certain genes. The accuracy requires one or more stable reference genes for calculating the gene expression levels using the 2^−ΔΔCq^ method [64]. Transcriptomic results suggest that there is always more than one gene stable in each set of samples, but none of them remains stable under all the conditions [5]. To date, *18S rRNA* [22, 28, 29], *actin* [25], *GAPDH* [27, 31], and *ubiquitin* [32] were used as reference genes in previous studies in spinach, although their stability was not validated yet.

One study in spinach seeds evaluated the stabilities of *GAPDH*, *actin*, *16*s *rRNA*, *TUBα*, and *18S rRNA*, and *18S rRNA* was taken as a relative stable reference gene [27]. In this report, we have determined *18S rRNA* as the optimal reference gene in roots, stems, leaves, flowers, and seedlings, as well as in leaves and roots in response to salinity stress.

*TUB* (*TUBα* or *TUBβ*) was commonly used as a reference gene in some plant species, such as *Raphanus sativus*, *Baphicacanthus cusia*, and *Hibiscus cannabinus* [33, 34, 57]. However, in this report, *TUBα* was regarded as the most unstable reference gene in spinach in many cases. This is similar to the situation in *Achyranthes bidentata* and Amaranthaceae. In *A. bidentata* under hormone treatments (e.g., indole-3-butytric acid and methyl jasmonate), NaCl, and drought, *TUB* was also determined to be an unstable reference gene when compared with other candidate references (e.g., *18S rRNA*, *actin*, *APT1*, *EF1α*, *GAPDH*, *TUBβ*, *UBC*, and *UBQ*) [35]. Besides, *EF1α* was regarded as the optimal reference gene in spinach under NaCl treatment. Similarly, *EF1α* was also the optimum gene in *A. bidentate* under NaCl and drought stresses [35]. It should be noted that *EF1α* was considered relatively unstable in several other species, such as *Silene vulgaris* and *Papaver rhoeas* [37, 57]. This implies that homologous genes in different plant species may not always exhibit similar expression levels under different conditions.

The recommended reference genes from NormFinder and BestKeeper look different (Table 2), because of their different mathematical models [11, 12]. BestKeeper uses Cq to calculate SD of candidate genes [11], but NormFinder uses 2^−ΔCq^ to calculate the expression stability [12]. In our results, the expression patterns of heat-responsive genes (*SobZIP9* or *SoHSFB2b*) are consistent when using the *SoARF* and *SoActin* as reference genes, which are recommended from NormFinder and BestKeeper, respectively (Figure 5). Importantly, the heat-responsive patterns of *SobZIP9* or *SoHSFB2b* normalized by *SoARF* and *SoActin* are also similar with previous reports [22], but these heat-responsive patterns are absolute opposite or exaggerated when using *TUBα* as a reference gene that is not recommended by BestKeeper and NormFinder. This indicates that the optimal genes obtained in response to heat stress by NormFinder and BestKeeper are reliable for normalization of gene expression.

Due to different expression levels of genes, the Cq values of some reference genes are high (Cq > 30, Figure 2), or the ΔCq between the reference gene and target genes is relatively large. In this condition, even if the amplification efficiency of both primers of the reference gene and target genes is close to 100%, the error of gene expression level is probably enlarged, due to using the 2^−ΔΔCq^ method [64, 70]. Thus, in this case, it is better to normalize the mRNA levels using the values of primer amplification efficiency rather than 100%.

In this study, optimal reference genes of each sample set were determined in spinach. *18S rRNA* and *ARF* were validated as internal reference genes in different organs. *Actin* and *ARF*, instead of *18S rRNA*, were the most suitable genes in leaves and roots under heat treatment. For reference genes under various abiotic stresses, *EF1α* and *ARF* were suitable for CdCl_2_, *18S rRNA* and *COX* for NaCl, *RPL2* and *actin* for NaHCO_3_, and *ARF* and *CYP* for Na_2_CO_3_. These results demonstrate that different reference genes should be used under different conditions [9]. Taken together, *actin* should be a reference gene for evaluating gene expression across all organs under various stress conditions, because *actin* has relatively better rank and *M*/SD values among all cases (Table 2).

## 5. Conclusion

It is important that optimal genes should be used for certain conditions when RT-qPCR is conducted to determine the normalized gene expression pattern using the 2^−ΔΔCq^ method. Commonly used housekeeping genes in plant species may not be suitable under all the conditions or in certain species. In this study, the optimal genes were determined for gene expression normalization in spinach organs and in response to stresses of heat, CdCl_2_, NaCl, NaHCO_3_, and Na_2_CO_3_. The data provide a list of useful reference genes for future studies of gene expression patterns in spinach.

## Figures and Tables

**Table 1:** Primers used in comparison of candidate reference genes in Spinach.

Gene name^1^	Gene ID^2^	Sequence of primer (forward/reverse)	Size of PCR product (bp)	Amplification efficiency (%)	Correlation coefficient (*R*^2^)
*18S rRNA*	*Spo14194*	GATTCCGACGAACAACTGCG	141	98.96	0.998
		AAGTAACATCCGCCGATCCC			
*Actin*	*Spo23599*	TGTTCACGACATCAGCCGAA	138	99.36	0.999
		CGTCGGGTAGCTCGTAGTTC			
*ARF*	*Spo09845*	CCGATAAGCTTGGCCTCCAT	125	92.53	0.997
		AGCCTTGCTAGCGATGTTGT			
*COX*	*Spo27102*	AGGTTGCTCATGCTGTCTTGA	168	94.85	0.991
		CAACGACACTGATCTGGCCT			
*CYP*	*Spo15438*	TCCTTTCCATGGCCAATGCT	132	93.11	0.999
		CCCTAACAACGTCCATGCCT			
*EF1α*	*Spo03008*	ACCTCTCAGGCTGATTGTGC	173	102.80	0.995
		GAGTACTTGGGAGTGGTGGC			
*GAPDH*	*Spo24687*	GGCTGCCATCAAGGAGGAAT	129	93.56	0.999
		GCAATTCCAGCCTTGGCATC			
*H3*	*Spo20638*	AAGAAGCCTCACCGTTACCG	178	94.48	0.998
		CCTCCTGAAGGGCCAAAACA			
*RPL2*	*Spo08157*	TTCTCGTCCGTCTCCCTTCT	198	101.18	0.997
		TACCCTCACCACCACCATGA			
*TUBα*	*Spo15071*	TAATGCCGCTGTTGCTACCA	137	94.90	0.998
		CTCTCTGCACCTTGGCAAGA			

^1^Full names of these 10 genes are *18S ribosomal RNA* (*18S rRNA*), *actin*, *ADP ribosylation factor* (*ARF*), *cytochrome c oxidase* (*COX*), *cyclophilin* (*CYP*), *elongation factor 1-alpha* (*EF1α*), *glyceraldehyde-3-phosphate dehydrogenase* (*GAPDH*), *histone H3* (*H3*), *50S ribosomal protein L2* (*RPL2*), and *tubulin alpha chain* (*TUBα*). ^2^These sequences and IDs were obtained from SpinachBase (http://www.spinachbase.org).

**Table 2:** The evaluation of candidate reference genes with NormFinder and BestKeeper^1^.

Method		Ranking order (better-good-average)									
	Rank^1^	1	2	3	4	5	6	7	8	9	10
	Organs/seedlings^2^										
NormFinder	Gene^3^	*ARF*	*EF1α*	*COX*	*GAPDH*	*RPL2*	*Actin*	*H3*	*18S rRNA*	*Tubα*	*CYP*
	**M1**	0.131	0.203	0.365	0.373	0.382	0.389	0.427	0.582	0.678	0.703
BestKeeper	Gene^3^	*18S rRNA*	*CYP*	*COX*	*Actin*	*RPL2*	*ARF*	*EF1α*	*H3*	*GAPDH*	*Tubα*
	SD	0.58	0.72	0.94	1.07	1.07	1.08	1.10	1.13	1.28	1.58
	Heat										
NormFinder	Gene^3^	*ARF*	*RPL2*	*EF1α*	*CYP*	*H3*	*GAPDH*	*COX*	*18S rRNA*	*Actin*	*Tubα*
	**M1**	0.212	0.228	0.238	0.387	0.424	0.454	0.500	0.757	0.850	0.928
BestKeeper	Gene^3^	*Actin*	*18S rRNA*	*GAPDH*	*RPL2*	*ARF*	*H3*	*EF1α*	*CYP*	*COX*	*Tubα*
	SD	0.57	0.60	0.77	0.92	0.93	0.95	1.10	1.11	1.16	1.67
	CdCl_2_										
NormFinder	Gene^3^	*CYP*	*RPL2*	*ARF*	*EF1α*	*H3*	*GAPDH*	*COX*	*Actin*	*18S rRNA*	*Tubα*
	**M1**	0.625	0.65	0.75	0.751	0.765	0.791	0.836	0.91	0.987	1.621
BestKeeper	Gene^3^	*ARF*	*EF1α*	*CYP*	*Actin*	*18S rRNA*	*RPL2*	*H3*	*GAPDH*	*COX*	*Tubα*
	SD	0.37	0.49	0.53	0.54	0.62	0.98	1.05	1.22	1.33	2.09
	NaCl										
NormFinder	Gene^3^	*EF1α*	*ARF*	*CYP*	*GAPDH*	*H3*	*COX*	*Actin*	*18S rRNA*	*RPL2*	*Tubα*
	**M1**	0.201	0.213	0.251	0.275	0.304	0.324	0.426	0.492	0.496	0.852
BestKeeper	Gene^3^	*18S rRNA*	*ARF*	*COX*	*EF1α*	*Actin*	*GAPDH*	*CYP*	*H3*	*RPL2*	*Tubα*
	SD	0.24	0.32	0.34	0.38	0.39	0.45	0.61	0.66	0.86	1.24
	NaHCO_3_										
NormFinder	Gene^3^	*RPL2*	*Actin*	*CYP*	*GAPDH*	*H3*	*COX*	*ARF*	*EF1α*	*Tubα*	*18S rRNA*
	**M1**	0.253	0.454	0.48	0.485	0.489	0.57	0.832	1.184	1.273	1.407
BestKeeper	Gene^3^	*Actin*	*RPL2*	*H3*	*GAPDH*	*COX*	*CYP*	*18S rRNA*	*ARF*	*EF1α*	*Tubα*
	SD	0.64	0.74	0.86	0.89	1.02	1.06	1.11	1.22	1.45	2.13
	Na_2_CO_3_										
NormFinder	Gene^3^	*ARF*	*H3*	*RPL2*	*CYP*	*EF1α*	*Actin*	*Tubα*	*18S rRNA*	*COX*	*GAPDH*
	**M1**	0.284	0.297	0.298	0.366	0.413	0.432	0.455	0.469	0.473	0.523
BestKeeper	Gene^3^	*COX*	*Actin*	*ARF*	*GAPDH*	*H3*	*Tubα*	*CYP*	*RPL2*	*EF1α*	*18S rRNA*
	SD	0.36	0.38	0.68	0.7	0.76	0.85	0.89	0.89	0.92	1.03

^1^Lower stability value **M1** obtained by NormFinder or SD obtained by BestKeeper indicates higher stability of housekeeping genes under each condition and is marked with bold text. ^2^These samples include leaves, stems, roots, flowers, and seedlings, as well as the leaves and roots under heat (37°C for 0, 1, 2, 4, 6, 8, 12, 24, and 48 h), heavy metal (200 *μ*M CdCl_2_ for 0, 1, 3, 6, 12, 24, and 48 h), salt (200 mM NaCl for 0, 1, 3, 6, 12, 24, and 48 h), and alkali (200 mM NaHCO_3_ or 100 mM Na_2_CO_3_, treated for 0, 1, 3, 6, 12, 24, and 48 h) treatments. Three biological replicates were taken for different organs/seedlings, as well as leaves and roots at each time point of all stress treatments. ^3^Names of candidate reference genes refer to Table 1.

## Data Availability

The data used to support the findings of this study are included in this published article and its supplementary information files.

## Supplementary Materials

Supplementary Materials Figure S1: specificity of primers used in RT-qPCR experiment. Figure S2: standard curves of ten candidate reference genes.
