# Supplementary material for: Selection and Validation of Reference Genes for RT-qPCR Analysis in Spinacia oleracea under Abiotic Stress
Source: Biomed Res Int. 2021 Feb 3;2021:4853632. doi: 10.1155/2021/4853632 (PMC7875621; doi:10.1155/2021/4853632)
Supplement: Supplementary Materials — Figure S1: specificity of primers used in RT-qPCR experiment. Figure S2: standard curves of ten candidate reference genes. [file 4853632.f1.pdf]

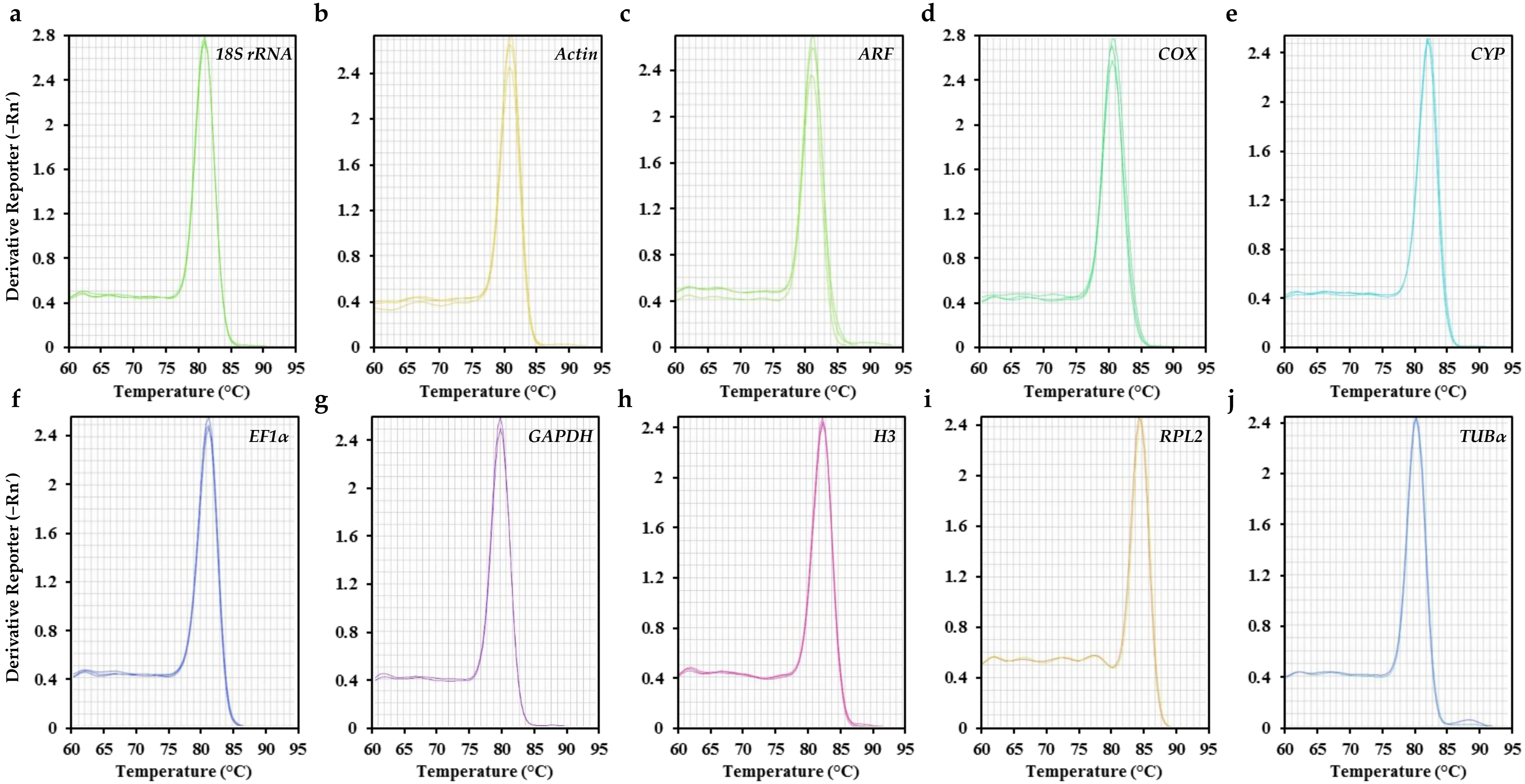

Figure S1. Specificity of primers used in RT-qPCR experiment. Melt Curves of each primer pairs were generated after amplification. Single peak represented high specificity of these primers. (a) *18S ribosomal RNA* (*18S rRNA*), (b) *actin*, (c) *ADP* (*ribosylation factor ARF*), (d) *cytochrome c oxidase* (*COX*), (e) *cyclophilin* (*CYP*), (f) *elongation factor 1-alpha* (*EF1*α), (g) *glyceraldehyde-3-phosphate dehydrogenase* (*GAPDH*), (h) *histone H3* (*H3*), (i) *50S ribosomal protein L2* (*RPL2*), (j) *tubulin alpha chain* (*TUB*α).

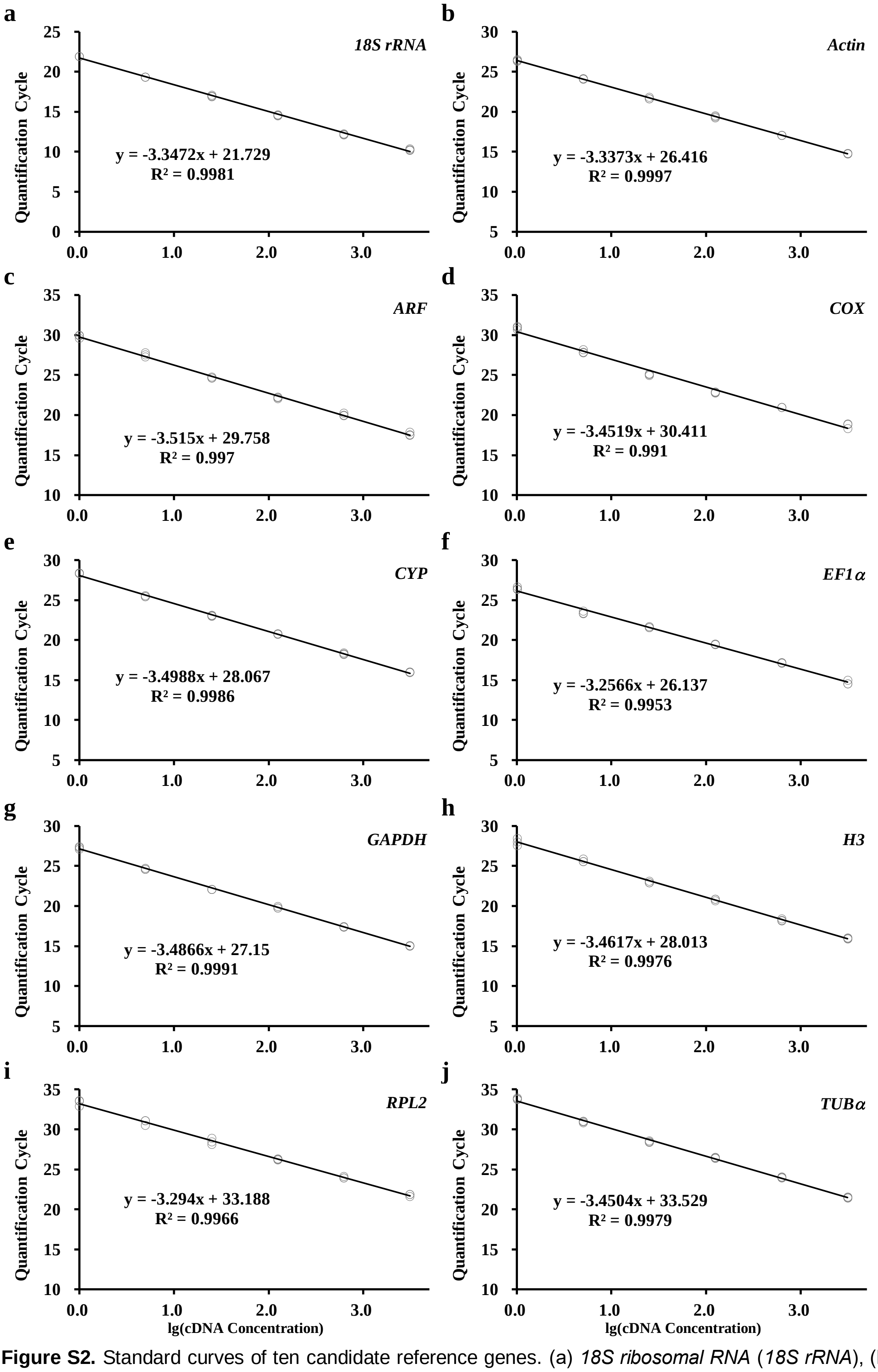

Figure S2. Standard curves of ten candidate reference genes. (a) *18S ribosomal RNA* (*18S rRNA*), (b) *actin*, (c) *ADP* (*ribosylation factor ARF*), (d) *cytochrome c oxidase* (*COX*), (e) *cyclophilin* (*CYP*), (f) *elongation factor 1-alpha* (*EF1α*), (g) *glyceraldehyde-3-phosphate dehydrogenase* (*GAPDH*), (h) *histone H3* (*H3*), (i) *50S ribosomal protein L2* (*RPL2*), (j) *tubulin alpha chain* (*TUBα*).
